# Costs associated with management of non-communicable diseases in the Arab Region: a scoping review

**DOI:** 10.7189/jogh.08.020410

**Published:** 2018-12

**Authors:** Shadi Saleh, Amena El Harakeh, Maysa Baroud, Najah Zeineddine, Angie Farah, Abla Mehio Sibai

**Affiliations:** 1 Department of Health Management and Policy, Faculty of Health Sciences, American University of Beirut, Beirut, Lebanon; 2 Refugee Research and Policy Program, Issam Fares Institute for Public Policy and International Affairs. American University of Beirut, Beirut, Lebanon; 3 Department of Epidemiology and Population Health, Faculty of Health Sciences, American University of Beirut, Beirut, Lebanon

## Abstract

**Background:**

Global mortality rates resulting from non-communicable diseases (NCDs) are reaching alarming levels, especially in low- and middle-income countries, imposing a considerable burden on individuals and health systems as a whole. This scoping review aims at synthesizing the existing literature evaluating the cost associated with the management and treatment of major NCDs across all Arab countries; at evaluating the quality of these studies; and at identifying the gap in existing literature.

**Methods:**

A systematic search was conducted using Medline electronic database to retrieve articles evaluating costs associated with management of NCDs in Arab countries, published in English between January 2000 and April 2016. 55 studies met the eligibility criteria and were independently screened by two reviewers who extracted/calculated the following information: country, theme (management of NCD, treatment/medication, or procedure), study design, setting, population/sample size, publication year, year for cost data cost conversion (US$), costing approach, costing perspective, type of costs, source of information and quality evaluation using the Newcastle–Ottawa Scale (NOS).

**Results:**

The reviewed articles covered 16 countries in the Arab region. Most of the studies were observational with a retrospective or prospective design, with a relatively low to very low quality score. Our synthesis revealed that NCDs’ management costs in the Arab region are high; however, there is a large variation in the methods used to quantify the costs of NCDs in these countries, making it difficult to conduct any type of comparisons.

**Conclusions:**

The findings revealed that data on the direct costs of NCDs remains limited by the paucity of this type of evidence and the generally low quality of studies published in this area. There is a need for future studies, of improved and harmonized methodology, as such evidence is key for decision-makers and directs health care planning.

Global mortality rates resulting from non-communicable diseases (NCDs) are reaching alarming levels with an increase from below 8 million between 1990 and 2010 to 34.5 million during year 2010 [1]. This figure is estimated to reach 52 million by 2030 [2,3]. Notably, low- and middle-income countries (LMICs) witnessed highest percentage increase of NCDs deaths with an expected average of 7 out of every 10 deaths occurring in developing countries by 2020 [4]. Eighty two percent of these deaths are caused by four major NCDs, namely cardiovascular diseases, chronic respiratory diseases (asthma and chronic obstructive pulmonary disease in particular), cancer, and diabetes [5-7]. Consistent with global trend, the Arab region was witnessing an increasing NCDs burden [8]. In Lebanon, 85% of deaths are attributed to NCDs [9,10], while in Morocco and Kuwait, NCDs account for 75% and 73% of deaths, respectively [11,12]. Furthermore, while deaths caused by infectious diseases are declining in the West, some countries in the region still carry a double burden of disease like Sudan, where 34% of deaths are attributable to NCDs, and 53% still result from communicable diseases [12,13]. The latter challenge of dealing with multiple diseases is intensified by several factors: limited human and financial resources, weak surveillance system, limited access to health care services and lack of financial protection in terms of insurance or public funding [14].

Worldwide, the rising burden of death and disability attributed to NCDs threatens the functionality and effectiveness of the health sector and imposes risks on economic stability and development of societies [15,16]. In several developed and developing countries, health costs and productivity loss associated with management of diabetes alone represent a significant share of gross domestic product (GDP), reaching 1% share from the US economy [17]. Economists are expressing major concerns about the long-term macroeconomic impact of NCDs on capital accumulation and GDP worldwide, with most severe consequences likely to be felt by developing countries [18]. In fact, it is estimated that NCDs costs will reach more than US$ 30 trillion in the coming two decades [19] further challenging the ability of health care systems to cope with these rising costs, especially in resource-scarce countries [18].

Considerable literature exists on economic evaluation and costs associated with NCDs in different regions worldwide, mostly in high-income countries (HICs) [20-23]. However, to date, no such studies exist in LMICs [4,24-27] and minimal effort was undertaken to synthesize and analyze current evidence addressing this issue in a comprehensive review [28-30]. Additionally, there has not been any attempt to collate and review relevant literature and evaluate the quality of existing studies on NCDs’ cost in the Arab region. This study aims to identify and synthesize available published evidence evaluating the cost associated with management and treatment of major NCDs across all Arab countries; to appraise critically these studies’ quality; and to identify the gap in existing literature. This study’s findings will aid in building a profile of the financial burden of NCDs in the Arab region, which would support and direct health care planning and future health research.

## METHODS

### Search strategy and inclusion criteria

**Figure 1:**
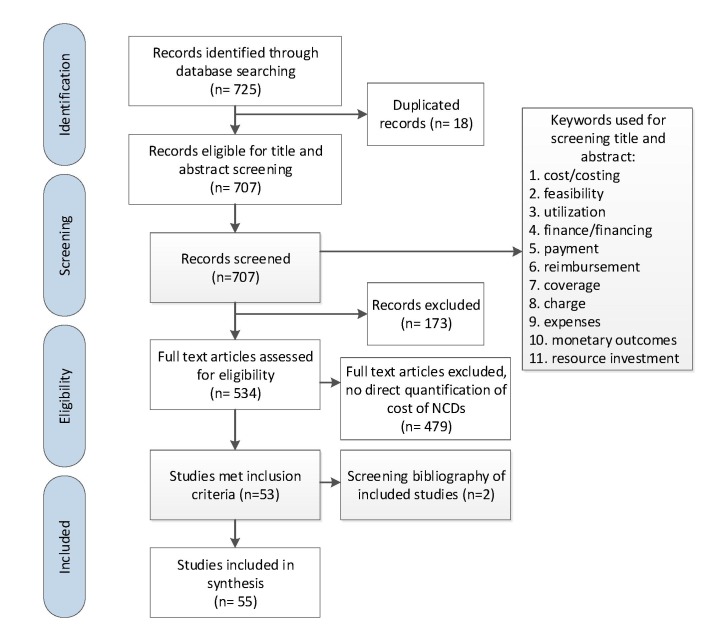
Flowchart of articles identified, included and excluded.

A systematic search was conducted using Medline electronic database to identify and retrieve articles evaluating the cost associated with management of NCDs in all 22 Arab countries; namely: Algeria, Bahrain, Comoros, Djibouti, Egypt, Iraq, Jordan, Kuwait, Lebanon, Libya, Mauritania, Morocco, Oman, Palestine, Qatar, Saudi Arabia, Somalia, Sudan, Syria, Tunisia, United Arab Emirates and Yemen. Based on their global economic burden on governments and populations, the following NCDs were selected: cardiovascular diseases, cancer, chronic respiratory diseases and diabetes [31]. Only papers published in English between January 2000 and April 2016 inclusive were included. The complete search strategy applied in this review is available in Appendix S1 of **Online Supplementary Document(Online Supplementary Document)**, and key inclusion and exclusion criteria are presented in **Figure 1**. The search strategy used MeSH terms and keywords relative to each of the four NCDs, their risk factors and costing including: Tobacco, Nutrition/Diet, Alcohol and Substance Abuse, Physical Inactivity, Hypertension, Cholesterol, Hyperlipidemia, Metabolic Syndrome, Salt and Sodium Intake, Diabetes, Cardiovascular disease, Cancer, Chronic Lung Dysfunction, Asthma, COPD, Renal Dysfunction, and Chronic Diseases, Health Care Costs, Health Expenditure, Health Resources, Insurance, Reimbursement, Fees, Charges, Feasibility Studies and Cost Benefit Analysis. The terms were combined with each of the 22 countries in the Arab region. Retrieved articles were screened and reviewed to assess their eligibility based on their content and study population. A total of 725 papers were identified to fit the initial search criteria. After removing duplicates, 707 papers remained for further screening.

### Study selection

Titles and abstracts of the initially identified articles were screened by two independent reviewers to assess whether they fulfill the selection criteria using keywords including cost/costing, feasibility, utilization, finance/financing, payment, reimbursement, coverage and charge, expenses, monetary outcomes and resource investment. Articles not including any of the above-mentioned keywords in the title or abstract were excluded. Hence, 534 articles were identified for full text review and were assessed by the two reviewers for relevance with regard to the research topic. Only those articles that provided direct quantification of costs associated with NCDs, their treatment, management, or risk factors within the target countries were included. Studies conducted outside of Arab region were excluded. Any disagreement between the two reviewers was resolved by discussion and consensus or through consultation with a third reviewer when needed. The identified eligible articles accounted for a total of 55 articles, tackling the issue of NCDs’ costs within at least one of the Arab countries.

### Data abstraction

Data was extracted from full texts included in this review using a data collection form composed of the following criteria (**Table 1**):

**Table 1:** Overview of the characteristics of the studies included in this scoping review*

Source	Year of publication/ year for cost data	Country		Study design	Sample size	NCD addressed	Source of data
Aďt-Khaled et al [32]	2000/1998	Algeria and Syria		Cross-sectional	10 countries	Asthma	Health system
Al Khaja et al [33]	2001/1998	Bahrain		Cross-sectional	3838 patients	Hypertension	Primary health care centers
Caro et al [34]	2002/1999	Egypt and Jordan		Cross-sectional	10 countries, 199 and 232 patients (from patient membership lists) from Egypt and Jordan respectively with patients less than 10 y old being the largest age group.	Thalassemia Major	Health system
Behbehani and Al-Yousifi [35]	2003/1996	Kuwait		Cross-sectional	36 (12 family and 24 non-family) primary health care centers	Asthma	Primary health care centers
Shaheen and Al Khader [36]	2005/2004	Kingdom of Saudi Arabia		Literature review	N/A	Chronic Renal Failure	N/A
Arevian [37]	2005/2002	Lebanon		Prospective follow up	375 patients		Primary health care center (socio-medical health center)
Elrayah et al [38]	2005/2003	Sudan		Descriptive cross-sectional	Diabetes		3 public and 3 private clinics
AlMarri [39]	2006/2002	Qatar		Cross-sectional analysis	Childhood diabetes mellitus type 1	Asthma	Health system
El-Zawahry et al [40]	2007/1999-2002	Egypt		Retrospective study	82 adult AML patients	Cancer – AML (acute myeloid leukemia)	Health system (National Cancer Institute)
Batieha et al [41]	2007/2003	Jordan		Cross-sectional	1711 patients	Chronic Renal Failure	Health system (56 hemodialysis units)
Abdel-Rahman et al [42]	2008/2003-2007	Jordan		Review	320 patients	Cancer	Cancer center
Ali et al [43]	2008/2007	Kingdom of Saudi Arabia		Prospective observational study & computer simulation model	598 patients	Diabetes	Health system
Dennison et al [44]	2008/2006	Sultanate of Oman		Cohort study	128 patients	Hematologic disorders	University hospital
El-Zimaity et al [45]	2008/2005-2008	Egypt		Cohort study	16 patients	Hematologic disorders	University Bone Marrow Transplant Unit
Strzelczyk et al [46]	2008/2006	Sultanate of Oman		Systematic Review	486 patients aged >13 years	Epilepsy	Hospital
Al-Naggar et al [47]	2009/2008	Yemen		Cross-sectional study	105 female doctors	Breast cancer	Four main hospitals in capital
Sabry et al [48]	2009/2008	Kingdom of Saudi Arabia		Cross-sectional study	23 adult chronic renal failure patients stabilized on hemodialysis	Chronic renal failure	Health system
Sweileh et. al [49]	2009/2006	Palestine		Descriptive study	95 patients	Cardiovascular (ischemic stroke)	Hospital
Shams & Barakat [50]	2010/2008		Egypt	Cross-sectional study	226 patients	Diabetes	University hospital
Al-Maskari [51]	2010/2005	United Arab Emirates		Cross-sectional study	150 patients	Diabetes	Two hospitals
Boutayeb et al [52]	2010/2007	Morocco		Cost analysis	N/A	Breast cancer	Country
Denewer et al [53]	2010/2007	Egypt		Cross-sectional study	5900 women	Breast cancer	Rural areas
Elrayah-Eliadarous et al [54]	2010/2005	Sudan		Cross-sectional study	822 patients	Diabetes	Public and private diabetes clinics
Valentine et al [55]	2010/2008	Kingdom of Saudi Arabia		Systematic Review	598 patients	Diabetes	Health system
Farag et al [56]	2011/2010	Egypt and Kingdom of Saudi Arabia		Review	NA	Diabetes	Health system
Osman et al [57]	2011/2009	Kingdom of Saudi Arabia		Prospective observational study	205 patients	Cardiovascular (ischemic heart disease)	Major cardiac center
Alameddine & Nassir [58]	2012/2010	Kingdom of Saudi Arabia		Retrospective study	516 patients	Bladder cancer	Medical center
Berraho et al [59]	2012/2009	Morocco		Cohort study	1978 new cases	Cervical cancer	Health system
Soliman & Roshd [60]	2012/2010	Egypt		Cross-sectional study	155 patients	End-stage renal disease	Nephrology centers
Tachfouti et al [61]	2012/2004	Morocco		Cross-sectional study	3500 new cases	Lung cancer	Health system
Al-Busaidi et al [62]	2013/2010	Sultanate of Oman		Cost analysis	91 646 adults and 55 426 children	Asthma	Health system
Algahtani et al [63]	2013/2010	Kingdom of Saudi Arabia		Prospective randomized clinical study	103 patients	Deep vein thrombosis	Tertiary care hospital
Alhowaish [64]	2013/2010	Kingdom of Saudi Arabia		Cross-sectional study	3.4 million patients	Diabetes	Health system
Almutairi and Alkharfy [65]	2013/2010-2011	Kingdom of Saudi Arabia		Retrospective observational study	300 patients	Diabetes	University hospital
Al-Rubeaan et al [66]	2013/2012	Kingdom of Saudi Arabia		Descriptive study	84 942 patients	Diabetes	Saudi National Diabetes Registry
Al-Sharayri et al [67]	2013/2012	Jordan		Cross-sectional study	556 prescriptions	Diabetes	Outpatient pharmacy in a medical center
Al-Shdaifat and Manaf [68]	2013/2010	Jordan		Cross-sectional study	175 patients	Chronic Renal Failure	3 Hospitals
Ghanname et al [69]	2013/2010	Morocco		Cost analysis	N/A	Asthma	Health system
Khadadah [70]	2013/2005	Kuwait		Cost analysis	93 923 adult patients and 70 158 children patients	Asthma	Health system
Alzaabi et al [71]	2014/2011	United Arab Emirates		Retrospective study	139 092 patients	Asthma	Health system
Ghanname et al [72]	2014/2010	Morocco		Cost analysis	N/A	Asthma	Health system
Lamri et al [73]	2014/2013	Algeria		Literature review	N/A	Diabetes	Health system
Mason et al [74]	2014/2010	Tunisia, Syria and Palestine		Cost-effectiveness analysis	N/A	Coronary heart disease	Health system
Isma'eel et al [75]	2012/2011	Lebanon, Bahrain, Jordan, Kuwait, Saudi Arabia, UAE and Oman		Descriptive study	N/A	Coronary heart disease	Health system
Younis et al [76]	2011/2008	Palestine		Cost analysis	N/A	Coronary heart disease	Tertiary care hospital
Shafie et al [77]	2014/2010	Algeria and Kingdom of Saudi Arabia		Cost-effectiveness analysis	279 and 901 respectively	Diabetes	Health system
Al-Busaidi et al [78]	2015/2013	Sultanate of Oman		Commentary	N/A	Asthma	Health system
Al-Kaabi & Atherton [79]	2015/2010	Qatar		Review	N/A	4 NCDs (cancer, cardiovascular, COPD and diabetes)	Health system
Antar et al [80]	2015/2011	Lebanon		Retrospective analysis	83 patients	Cancer (multiple myeloma)	Tertiary care hospital
Eltabbakh et al [81]	2015/2011	Egypt		Prospective, single-center cohort study	1286 patients	Liver cirrhosis	Tertiary care hospital
Gupta et al [82]	2015/2013	Kingdom of Saudi Arabia		Cost-effectiveness analysis	680 patients	Diabetes	Health system
Home et al [83]	2015/2013	Algeria		Cost-effectiveness analysis	473 patients	Diabetes	Health system
Schubert et al [84]	2015/2015	United Arab Emirates		Network meta-analysis	N/A	Diabetes	Health system
Thaqafi et al [85]	2015/2015	Kingdom of Saudi Arabia		Cost analysis	N/A	Hematologic cancer	Health system
Ahmad et al [86]	2016/2014	Sultanate of Oman		Retrospective study	50 adult cardiac arrest patients who had undergone CPR	Cardiac arrest	Hospital

CPR – cardiopulmonary resuscitation, NCD – non-communicable disease, COPD– Chronic obstructive pulmonary disease

*N/A refers to not applicable whereby the data of interest is not specified in the respective reference.

- Country – based on study location;
- Category – based on main theme/topic addressed: management of the NCD, treatment/medication, or procedure;
- Study design – classified as cross-sectional, cohort, review, or systematic review/meta-analysis;
- Setting – described as being a health system, cases from primary healthcare center, hospital, or clinic (private vs. public);
- Population/Sample size;
- Year of publication;
- Year for cost data;
- Costing approach – classified as bottom up or top down;
- Costing perspective – classified as societal, governmental, provider or patient;
- Type of costs – classified as direct medical, indirect medical and indirect;
- Source of information – classified as survey, medical record, health information survey or electronic database.

The findings are presented by type of NCD. US$ were used when assessing economic costs across all studies to enhance comparability. Other reported local currencies were converted to US$ based on the exchange rate specified by the corresponding study. When exchange rate was not mentioned, conversion to US$ was performed using the conversion rate specific to the year of publication of the study.

### Quality evaluation

The quality of included cross-sectional, case-control and cohort studies was evaluated using the Newcastle–Ottawa Scale (NOS), which is based on three domains: selection, comparability and exposure [87]. A maximum of one star can be awarded to each question in the selection category and one star to each question included in the exposure category, while a maximum of two stars can be awarded to a single question in the comparability section. For each study, a quality score is then generated by adding up the number of stars given and would not exceed 9 stars. The modified version of the NOS used for descriptive and cross-sectional studies was adopted from the systematic review conducted by Jaspers et al (2015) [88].

## RESULTS

We initially identified 725 potentially eligible references published between 2000 and 2016 (**Figure 1**). Of those, and after title and abstract and full text screenings, 55 studies met the inclusion criteria and were thoroughly described in the review.

### Overview of included studies

The reviewed articles covered most of the Arab region, yet no data was available from 6 of the 22 Arab countries, namely Iraq, Somalia, Libya, Mauritania, Djibouti and Comoros. The majority of studies (n = 27) originated from high-income Arab countries, while 19 were conducted in lower-middle income and 12 were from upper-middle income Arab countries. This reflected GDP variation across the reviewed articles. Most studies were conducted in the Kingdom of Saudi Arabia (n = 15), Egypt (n = 8) and Jordan (n = 7) whereas 5 studies were conducted in multiple countries (**Table 1**). Included studies were mainly observational with retrospective or prospective design, few other studies were modeling, reviews, systematic reviews, meta-analyses, commentaries and cost analyses. In 30 studies, the setting represented was the health system. The remaining studies sampled eligible participants from hospitals (n = 15), medical centers (n = 5), primary health care centers (n = 3) and private and public clinics (n = 2) (**Table 1**).

The most frequently studied NCD was diabetes (n = 18) whereas chronic respiratory diseases (mainly asthma, n = 9) and cancer were each analyzed in 11 studies. Twelve studies focused on cost associated with management of cardiovascular diseases while 7 studies focused on other NCDs mainly chronic renal failure (**Table 1**). Only one study addressed the four NCDs together.

All of the included studies reported direct medical costs associated with the management of the four major non-communicable diseases in the Arab region. Some studies (n = 15) also included indirect costs such as loss of productivity and premature death. While only one article described direct non-medical costs that are not directly related to medical services such as transportation. (**Table 4**).

**Table 4:** Results indicating cost associated with the management of other NCDs reported in the included studies

Source	Country	Addressed NCD	Population studied /contacted	Category/ Costing Scope	Outcome specified as	Point estimate (in US$)	Quality score
Shaheen and Al Khader [36]	Kingdom of Saudi Arabia	Chronic renal failure	NA	Procedure	Annual cost incurred toward maintenance hemodialysis	19 400	NA
Batieha et al [41]	Jordan	Chronic renal failure	Patients on hemodialysis	Procedure	Total annual cost of hemodialysis including hemodialysis sessions, medications and investigations, admissions and arterial access	29 715 553	4
Strzelczyk et al [46]	Sultanate of Oman	Epilepsy	Patients aged >13 years old	Management	% attributed to inpatient admission	52%	NA
Sabry et al [48]	Kingdom of Saudi Arabia	Chronic renal failure	Adult chronic renal failure patients stabilized on hemodialysis	Treatment	Mean cost of 6 mo use of (1) tinzaparin sodium per patient compared to that of (2) unfractionated heparins	(1) 67.57 and (2) 51.23	2
Soliman & Roshd [60]	Egypt	End-stage renal disease	Chronic renal failure patients	Management	(1) annual cost for thrice-weekly hemodialysis, (2) cost of CAPD catheter insertion, (3) annual cost of 3 to 4 fluid exchanges, (4) costs for pre-transplantation and transplantation procedures, (5) annual costs for immunosuppressive drugs	(1) 3250, (2) 150, (3) [4500-6000], (4) 6000-7500 and (5) 3250-6000	1
Al-Shdaifat and Manaf [68]	Jordan	Chronic renal failure	Chronic renal failure patients	Procedure	(1) total annual cost at MOH and (2) annual cost per patient	(1)17.7 million and (2) 9976	3
Eltabbakh et al [81]	Egypt	Liver cirrhosis	Liver cirrhosis patients	Procedure	Annual cost of detecting a treatable HCC case by (1) ultrasound and (2) by both ultrasound and AFP	(1) 560 and (2) 650	2

MOH – Ministry of Health, HCC – Hepatocellular carcinoma, AFP –Alpha-fetoprotein, CAPD – Continuous ambulatory peritoneal dialysis

*N/A refers to “not applicable” whereby the data of interest is not specified in the respective reference.

Cost data collected through surveys represented the most commonly used data source (n = 19) while 12 studies relied on data retrieved from health information systems of ministries, hospitals and insurance companies followed by prior estimates published in the literature, which is represented as electronic database (n = 12) in **Table 5**. Medical records were used in eight studies and a data source was not applicable for the component costs of one study. Some studies included several cost components and data sources without giving a clear description of which data sources were used for particular components.

**Table 5:** Results indicating costing approach, costing perspective, type of costs and sources of information associated with the management of the NCDs reported in the included studies*

Source	Year	Costing approach	Costing perspective	Type of costs	Sources of information*
Aďt-Khaled et al [**32**]	2000	Bottom up	Governmental	Direct medical and indirect	Survey
Al Khaja et al [**33**]	2001	Bottom up	Societal	Direct medical	Survey
Caro et al [**34**]	2002	N/A	Patient	Direct medical and indirect	Survey
Behbehani and Al-Yousifi [**35**]	2003	Top down	Provider	Direct medical	Survey
Shaheen and Al Khader [**36**]	2005	N/A	Governmental	Direct medical	NA
Arevian [**37**]	2005	N/A	Provider	Direct medical and indirect	Medical record
Elrayah et al [**38**]	2005	Bottom up	Provider	Direct medical and indirect	Survey
Al Marri [**39**]	2006	Bottom up	Provider	Direct medical	Health information system
El-Zawahry et al [**40**]	2007	Bottom up	Patient	Direct medical	Medical record
Batieha et al [**41**]	2007	Bottom up	Patient	Direct medical	Survey
Abdel-Rahman et al [**42**]	2008	Bottom up	Provider	Direct medical	Medical record
Ali et al [**43**]	2008	Bottom up	Provider	Direct and indirect medical cost	Survey
Dennison et al [**44**]	2008	Top down	Provider	Direct medical	Medical record
El-Zimaity et al [**45**]	2008	N/A	Patient	Direct medical	Medical record
Strzelczyk et al [**46**]	2008	Bottom up	Patient	Direct medical and indirect	Electronic databases
Al-Naggar et al [**47**]	2009	N/A	Provider	Direct medical	Survey
Sabry et al [**48**]	2009	N/A	Patient	Direct medical	Survey
Sweileh et. al [**49**]	2009	Bottom up	Patient	Direct medical	Survey
Shams & Barakat [**50**]	2010	N/A	Patient	Direct medical and indirect	Survey
Al-Maskari [**51**]	2010	Bottom up	Patient	Direct medical	Survey
Boutayeb et al [**52**]	2010	Bottom up	Provider	Direct medical	Secondary data
Denewer et al [**53**]	2010	Bottom up	Patient	Direct medical	Survey
Elrayah-Eliadarous et al [**54**]	2010	Top down	Patient	Direct medical	Survey
Valentine et al [**55**]	2010	Bottom up	Provider	Direct medical	Electronic databases
Farag et al [**56**]	2011	Bottom up	Provider	Direct medical	Electronic databases
Osman et al [**57**]	2011	Bottom up	Provider	Direct medical	Medical record
Alameddine & Nassir [**58**]	2012	Top down	Provider	Direct medical	Medical record
Berraho et al [**59**]	2012	Bottom up	Patient	Direct medical	Health information system
Soliman & Roshd [**60**]	2012	Bottom up	Patient	Direct medical	Survey
Tachfouti et al [**61**]	2012	Bottom up	Governmental	Direct medical	Health information system
Al-Busaidi et al [**62**]	2013	Bottom up	Patient	Direct medical	Electronic databases
Algahtani et al [**63**]	2013	Bottom up	Provider	Direct medical	Health information system
Alhowaish [**64**]	2013	Top down	Governmental	Direct medical	Health information system
Almutairi and Alkharfy [**65**]	2013	Bottom up	Governmental	Direct medical	Health information system
Al-Rubeaan et al [**66**]	2013	Bottom up	Governmental	Direct medical	Health information system
Al-Sharayri et al [**67**]	2013	Bottom up	Provider	Direct medical	Medical record
Al-Shdaifat and Manaf [**68**]	2013	Bottom up and top down	Provider	Direct medical and nonmedical and indirect	Health information system
Ghanname et al [**69**]	2013	Bottom up	Patient	Direct medical	Health information system
Khadadah [**70**]	2013	Bottom up	Patient	Direct medical	Survey
Alzaabi et al [**71**]	2014	Bottom up	Government	Direct medical	Health information system
Ghanname et al [**72**]	2014	Bottom up	Patient	Direct medical	Health information system
Lamri et al [**73**]	2014	Top down	Patient	Direct medical and indirect	Electronic databases
Mason et al [**74**]	2014	Top down	Governmental and Provider	Direct medical and indirect	Survey
Younis et al [**76**]	2011	N/A	Provider	Direct medical	Health information system
Isma'eel et al [**75**]	2012	N/A	Patient	Direct medical	Electronic databases
Shafie et al [**77**]	2014	Bottom up	Patient	Direct medical and indirect	Survey
Al-Busaidi et al [**78**]	2015	Bottom up	Patient	Direct medical	Electronic databases
Al-Kaabi & Atherton [**79**]	2015	Top down	Societal	Direct medical and indirect	Electronic databases
Antar et al [**80**]	2015	Bottom up	Provider	Direct medical	Health information system
Eltabbakh et al [**81**]	2015	Bottom up	Patient	Direct medical and indirect	Survey
Gupta et al [**82**]	2015	Bottom up	Societal	Direct medical and indirect	Electronic database
Home et al [**83**]	2015	Bottom up	Societal	Direct medical and indirect	Electronic database
Schubert et al [**84**]	2015	Bottom up	Provider	Direct medical	Electronic database
Thaqafi et al [**85**]	2015	Bottom up	Provider	Direct medical	Electronic database
Ahmad et al [**86**]	016	Top down	Patient	Direct medical	Health information system

*N/A refers to “not applicable” whereby the data of interest is not specified in the respective reference.

Among the 55 studies included, 23 (42%) studies described the patient’s perspective and 21 (38%) studies described the provider’s perspective in estimating the costs highlighting that the majority of the studies focused on the costs that fall on either patients or health care institutions providing health services. Eight studies looked at the governmental costs associated with NCDs. The remaining studies (n = 8) described the societal level costs.

Although most of the studies did not clearly indicate the costing approach used, the overall aim of the cost analysis and the sources of data assisted in determining the costing approaches followed. Most of the studies (n = 36) estimated the costs using a bottom up approach or micro-costing, while only nine studies relied on a top-down approach or gross-costing in their measurements. Only one study reported using both approaches, while identifying the costing approach was not applicable in seven of the included studies.

### Quality of the included studies

The majority of the studies were appointed a quality score (34 of the 55 included studies). In the studies where a quality score was not assigned, the study design and methodology made quality assessment not feasible. The median quality score over all the studies was three out of nine (interquartile range 2-4). Two thirds of the eligible and scored studies scored three points or less, showing that most of the studies were of low to very low quality.

### Cardiovascular diseases

As part of a cost-effectiveness analysis by Mason et al (2014) for the implementation of salt reduction policies [74], health care cost of coronary heart diseases (CHD) in Palestine was estimated (**Table 2**). The calculation of health care cost of CHDs incorporated standardized unit cost per patient for a number of CHD conditions, namely, acute myocardial infractions (AMI), secondary prevention following AMI, unstable angina, chronic heart failure (treated in a hospital setting, or in the community), and hypertension [74]. Healthcare cost of coronary heart diseases in Palestine was estimated to be US$ 354 719 519 [74] (**Table 2**).

**Table 2:** Results indicating cost associated with the management of the cardiovascular diseases and cancer reported in the included studies

Source	Country	Addressed NCD	Population studied/contacted	Category/ Costing Scope	Outcome specified as	Point estimate (in US$)	Quality score
**Cardio-vascular diseases:**							
Al Khaja et al [33]	Bahrain	Hypertension	Patients with uncomplicated essential hypertension	Medication	Monthly cost of an antihypertensive drug (indapamine)	7.7	4
Caro et al [34]	Egypt and Jordan	Thalassemia major	Patients or their caregivers if less than 14 years old	Management	(1) % of hospitalized patients with a mean LOS of 10 days during the past 6 months, (2) days missed from employment and (3) days missed from school during 1 month	(1) 20%, (2) 2 days and (3) 3 days	3
Dennison et al [44]	Sultanate of Oman	Hematologic disorders	Patients who need hematopoietic stem cell transplant	Procedure	Approximate cost per uncomplicated transplant	50 000	2
El-Zimaity et al [45]	Egypt	Hematologic disorders	Patients with chronic or acute myeloid leukemia, aplastic anemia, acute lymphoblastic leukemia or aggressive lymphoma	Procedure	Average estimate cost per transplant	8446	1
Sweileh et. al [49]	Palestine	Ischemic stroke	Stroke patients	Treatment (therapy and medications)	Average monthly cost for treatment of post-stroke complications	52	6
Osman et al [57]	Kingdom of Saudi Arabia	Ischemic heart disease (IHD)	Patients diagnosed or suspected to have IHD	Management	Average direct cost (medication, hospital bed use and procedure) per patient	10 710	4
Algahtani et al [63]	Kingdom of Saudi Arabia	Deep vein thrombosis	Symptomatic adult patients with acute proximal DVT of the lower limbs	Treatment	Mean outpatient treatment cost per case	1750	3
Ahmad et al [86]	Sultanate of Oman	Cardiac arrest	>18 y old who had cardiac arrest, received at least one attempt at CPR and were potential DNR candidates	Management	Average cost of post-resuscitation care per patient including cost of medications, laboratory investigations, imaging, minor procedures and hospital stay in ICU or HDU	1958.9	5
Al-Kaabi & Atherton [79]	Qatar	Cardiovascular diseases	NA	Treatment	Total direct and indirect cost including personal medical; non-medical costs, and income losses	863 billion	NA
Mason et al [74]	(1) Tunisia, (2) Syria and (3) Palestine	Coronary heart disease	NA	Management	The total cost saving of having a combination of 3 salt-reduction policies	(1) 39 000 000, (2) 6 000 000 & (3) 1 300 000	NA
Isma'eel et al [75]	(1) Lebanon, (2) Bahrain, (3) Jordan, (4) Kuwait, (5) Saudi Arabia, (6) UAE and (7) Oman	Cardiovascular event	Public	Treatment	Cost of treatment using 3 types of statins to prevent 1 CV event in 5 years	(1) 79 388-105 589, (2) 81 505-190 530, (3) 109 578-112 348, (4) 122 786-202 147, (5) 81 323-122 786, (6) 113 260-217 203, (7) 111 143-202,575	1
Younis et al [76]	Palestine	Cardiac catheterization	N/A	Procedure	Total cost of unit (medical equipment, salaries, overhead costs, and variable costs)	613 544.63	NA
**Cancer:**							
El-Zawahry et al [40]	Egypt	Acute myeloid leukemia	Adult AML patients	Treatment	Median total cost of conventional chemotherapy per case	5817	3
Abdel-Rahman et al [42]	Jordan	Mainly leukemia, nonmalignant hematological disorders and thalassemia major	Transplant patients	Procedure	Average charge of (1) autologous and (2) allogeneic transplants	(1) 35 067 and (2) 66 438	NA
Al-Naggar et al [47]	Yemen	Breast cancer	Female OBGYN doctors	Procedure	% of doctors who do not send asymptomatic women for screening	23.8% (25 doctors)	NA
Boutayeb et al [52]	Morocco	Breast cancer	NA	Treatment	Total cost of breast cancer chemotherapy per case	13 360	NA
Denewer et al [53]	Egypt	Breast cancer	Women in rural areas	Treatment	(1) cost of screening per cancer case, (2) total cost of treatment for screened cases	(1) 415 and (2) 1015-1215	3
Alameddine & Nassir [58]	Kingdom of Saudi Arabia	Bladder cancer	Suspected urothelial cancer patients	Procedure	Total cost of 563 urine cytology tests	37 533	4
Berraho et al [59]	Morocco	Cervical cancer	New cases	Management	Total cost of care one year after diagnosis	13 589 360	2
Tachfouti et al [61]	Morocco	Lung cancer	New cases	Management	Total medical cost	12 million	3
Thaqafi et al [85]	Kingdom of Saudi Arabia	Hematological cancer	Patients with prolonged neutropenia or undergoing bone marrow or hematopoietic stem-cell transplantation	Medication	Estimated cost of alternative interventions (1) voriconazole, (2) LAMB, and (3) caspofungin.	(1) 7654, (2) 16 564 and (3) 17 362	N/A
Antar et al [80]	Lebanon	Multiple myeloma	Patients with multiple myeloma performing consecutive hematopoietic stem cell mobilization attempts	Procedure	Average cost of (1) chemo-mobilizing and (2) G-CSF and preemptive plerixafor mobilization strategies	(1) 7536 and (2) 7886	4
Al-Kaabi & Atherton [79]	Qatar	Cancer	NA	Management	Total direct and indirect cost including personal medical; non-medical costs, and income losses	290 billion in 2010 expected to reach 458 billion in 2030	N/A

IHD – ischemic heart disease, CV – cardiovascular, DNR – do not resuscitate, AML – acute myeloid leukemia, LAMB – Liposomal Amphotericin B

*N/A refers to “not applicable” whereby the data of interest is not specified in the respective reference.

A second study from Palestine also quantified costs associated with treating cardiovascular diseases; more specifically, the study estimated total cost of the cardiac catheterization unit in a major governmental hospital in Palestine as part of cost-volume-profit analysis [76]. Total cost calculations included fixed costs of medical equipment, furniture and other equipment, staff salaries, and overhead costs, and variable costs related to type of patient diagnosis, and respective procedures. Total unit cost was found to be US$ 613 544.63, with greatest costs attributed to variable costs of catheterization unit [76].

Isma’eel et al (2011) estimated the cost to the public of preventing a single cardiovascular event focusing on statins in seven Arabic countries and those are Lebanon, Bahrain, Jordan, Kuwait, Saudi Arabia, UAE and Oman [75]. The study compared cost based on defined daily dose, and compared costs of using one of three different statins for prevention. For instance, in Lebanon, the cost to the public was found to range between US$ 79 388 and US$ 105 589, depending on the statin used for treatment. In Bahrain, the cost to the public to prevent one cardiovascular event using statins ranged between US$ 81 505 and US$ 190 530. Conversely, in Kuwait, the estimated cost to the public ranged between US$ 122 786 and US$ 202 147, depending on the statin used for treatment [75].

### Cancer

Three studies quantified total costs associated with treating or managing cancer (breast, lung, or cervical) to Moroccan health care authorities for up to one year after diagnosis (**Table 2**). Boutayeb et al (2010) estimated total cost of breast cancer treatment by chemotherapy for patients in early stages of breast cancer to be between US$ 13 300 000 and US$ 28 600 000, based on international guidelines [52]. The upper bound estimation assumes all new cancer cases are treated. These costs were calculated by estimating the number of women in Morocco with breast cancer, and took into consideration alternative treatment protocols, per unit and per whole cycle [52]. Tachfouti et al (2012) conducted similar calculations to quantify direct costs of managing lung cancer in Morocco [61]. Taking into consideration the incidence of lung cancer, by stage, in the Moroccan population, also, taking into consideration treatment protocols as per international guidelines for each stage of lung cancer, the authors estimated that total medical costs of lung cancer are approximately US$ 12 000 000 [61]. Berraho et al (2012) used a similar methodology to Tachfouti et al (2012) to calculate total costs of managing cervical cancer in Morocco [59,61]. After estimating the incidence of cervical cancer cases, by stage, in the Moroccan population, and costs of management based on whole-cycle sets, the authors estimated total cost of cervical cancer care to be US$ 13 589 360.

### Diabetes mellitus

Elrayah et al (2005) calculated annual direct costs to diabetic children attending public and private diabetes clinics in Sudan, that were associated with controlling diabetes mellitus type 1 [54] (**Table 3**). The authors estimated the annual direct cost per diabetic child to be US$ 283 including costs of insulin, blood and urine tests and hospital admission and doctors’ fees. In 2010, the authors conducted a survey to determine out-of-pocket contributions made by patients with diabetes mellitus type 2 on ambulatory care and medications used to control diabetes, and found that annual direct cost per patient was approximately US$ 175. Patients aged 65 years and older made the greatest out-of-pocket contributions; furthermore, patients receiving ambulatory outpatient care at private clinics paid significantly more for clinic visits compared to patients receiving care at public facilities [54].

**Table 3:** Results indicating cost associated with the management of diabetes and chronic respiratory diseases reported in the included studies*

Source		Country		Addressed NCD	Population studied/ contacted	Category/costing scope	Outcome specified as	Point estimate (in US$)	Quality score
**Diabetes:**									
Arevian [37]		Lebanon		Diabetes	Diabetic patients	Management	Annual direct health care cost per a fully complaint case	125 compared to 481 in a tertiary care center	2
Elrayah et al [34]		Sudan-Khartoum		Diabetes	Parents of diabetic children	Management	Annual direct cost per case (including insulin, blood and urine tests, hospital admission and doctors' fee)	283	1
Ali et al [43]		Kingdom of Saudi Arabia		Diabetes	Patients with diabetes that were inadequately controlled on their current therapy of human insulin	Treatment	(1) annual direct cost of diabetes, (2) direct medical cost savings per patients for conversion from human insulin to BIAsp 30 therapy	(1) 400-700 million and (2) 14 547	3
Shamsa & Barakat [50]		Egypt		Diabetes	Patients with diabetes >18 years old	Treatment	Rate of adherence to medication based on the relation between cost (direct and indirect) and income	57.7% when relation was adequate, 24.8% when relation was not adequate	6
Al-Maskari [51]		United Arab Emirates		Diabetes	Patients with diabetes	Management	Total annual direct cost of DM (1) without and (2) with (macro and microvascular) complications per case	(1) 1605 and (2) 15 104	4
Elrayah-Eliadarous et al [54]		Sudan		Diabetes	Patients with diabetes >30 y old with a diabetes duration of 1-5 years	Management	Average annual direct cost (ambulatory care and drugs) of diabetes control per case	175	3
Valentine et al [55]		Kingdom of Saudi Arabia	Diabetes		Patients with diabetes that were inadequately controlled on their current therapy of human insulin	Treatment	Difference in direct cost between BIAsp and human insulin	15,786	NA
Farag et al [56]		Egypt & Kingdom of Saudi Arabia	Diabetes		NA	Management	Percentage of the country's total health expenditure	16% for Egypt and 21% for KSA	NA
Alhowaish [64]		Kingdom of Saudi Arabia	Diabetes		Diabetic patients	Management	Total annual national health expenditure	0.9 billion	2
Almutairi and Alkharfy [65]		Kingdom of Saudi Arabia	Diabetes		Diabetic patients	Management	Total annual direct medical cost (drug therapy, diagnostic procedures, hospitalization and outpatient visits)	1,384.19 for HbA1c <7%; 2036.11 for HbA1c 7%-9%, and 3104.86 for HbA1c >9%	NA
Al-Rubeaan et al [66]		Kingdom of Saudi Arabia	Diabetes		Diabetic patients	Medication	Annual insulin cost per patient for (1) Diabetes, (2) DM2 and (3) gestational diabetes	(1) 308, (2) 375 and (3) 267	4
Al-Sharayri et al [67]		Jordan	Diabetes		Patients on (1) traditional vials or (2) cartridges	Medication	Average direct cost per patient	(1) 7.31 and (2) 31.18	2
Schubert et al [84]		United Arab Emirates	Diabetes		Diabetic patients	Medication	Cost of canagliflozin (1) 100 and (2) 300 mg equivilant to cost of reaching HbA1c <7% with dapagliflozin 10 mg per day	(1) 2.11 and (2) 2.45	NA
Home et al [83]		Algeria	Diabetes		Patients with diabetes starting insulin detemir	Medication	Direct cost per patient simulated over 30 y with (1) insulin detemir compared to (2) OGLDs alone	(1) 15 782 vs (2) 10 563	NA
Gupta et al [82]		Kingdom of Saudi Arabia		Diabetes	Patients with diabetes	Management	Total direct cost (treatment, management and complication) of switching from (1) biphasic human insulin 30, (2) insulin glargine to biphasic insulin aspart 30	(1) 53 128-53 575 and (2) 61 569-52 849	NA
Al-Kaabi & Atherton [79]		Qatar		Diabetes	NA	Management	Total direct and indirect cost including personal medical; non-medical costs, and income losses	500 billion in 2010, expected to reach 745 billion in 2030	NA
Shafie et al [77]		(1) Algeria & (2) Kingdom of Saudi Arabia		Diabetes	Patients with diabetes	Management	Total cost (treatment, management and complication) of switching from glucose lowering drugs only to it coupled with biphasic insulin aspart 30 per patient	(1) 11 880 to 16 831 and (2) 51 158 to 49 263	NA
Lamri et al [73]		Algeria		Diabetes	NA	Management	Total annual spending on diabetes care for the health system	513 million	NA
**Chronic respiratory diseases:**									
Aďt-Khaled et al [32]		Algeria and Syria		Asthma	Pharmacies	Treatment (long term)	Annual cost per a persistent mild, moderate or severe case	32, 52 and 92 respectively in Algeria; 104 for a moderate case in Syria	2
Behbehani and Al-Yousifi [35]		Kuwait		Asthma	Heads of primary health care centers	Medications	Annual cost per a moderate case (using inhaled steroids and short-acting beta-agonists only)	562	3
AlMarri [39]		Qatar		Asthma	Asthma hospitalized patients	Hospital admission	Average cost per admission	1544	3
	Sultanate of Oman			Asthma	Asthma patients	Treatment	Total annual direct cost of treatment including medications	159 900 761	NA
	Morocco			Asthma	Individuals purchasing anti-asthmatic drugs	Treatment	Average monthly cost of anti-asthmatic treatment between 1999 and 2010	[16.42-12.36]	NA
	Kuwait			Asthma	Patients (adults and children) with asthma	Treatment	Total annual direct cost of treatment including outpatient, emergency and inpatient visits and medications	208 244 564	NA
Al-Kaabi & Atherton [79]	Qatar			COPD	NA	Treatment	Total direct and indirect cost including personal medical; non-medical costs, and income losses	2.1 trillion in 2010 expected to reach 4.8 trillion in 2030	NA
Al-Busaidi et al [78]	Sultanate of Oman			Asthma	NA	Management	Total annual cost of asthma management	159 741 021	NA
Ghanname et al [72]	Morocco			Asthma	NA	Medications	Total annual cost of anti-asthmatic drugs	24 361 920	NA
Alzaabi et al [71]	United Arab Emirates			Asthma	Asthma patients	Treatment	Total direct cost of per patient mainly outpatient visits	207	2

*N/A refers to “not applicable” whereby the data of interest is not specified in the respective reference.

A smaller scale study from Lebanon [37], conducted at a primary health care center in Beirut, estimated the direct cost of treating a fully compliant patient with diabetes mellitus type 2 to be US$ 125 (**Table 3**). Direct cost calculations included costs of physician services, laboratory tests, drugs, inpatient care and emergency visits. Cost per patient attending the primary health care center was found to be lower than the estimated direct health care cost of US$ 481 for a fully compliant diabetes mellitus type 2 patient attending private clinics at a tertiary medical care center in Lebanon.

In a national cross-sectional survey conducted in Saudi Arabia, Alhowaish (2013) estimated the total annual national health expenditure to be US$ 0.9 billion, which represents around 21% of the country’s total health expenditure [56,64]. This figure is not restricted to only direct medical costs associated with management of diabetes in Saudi Arabia. Another study examined annual direct costs of diabetes at the national level and estimated the amount to be between US$ 400 to 700 million [43]. In comparison, a study from Qatar showed that direct and indirect medical cost of diabetes management, including personal medical expenses, nonmedical costs and income losses reached US$ 500 billion in 2010 and projections showed an expected rise to US$ 745 billion in 2030 due to several factors [79].

### Asthma

Two studies from Kuwait quantified costs associated with treating asthma (Table 3). The first determined the annual cost of asthma medications, based on severity, while the second evaluated direct costs of treating asthma at the national level and determined direct costs associated with emergency department visits, outpatient clinic visits, and asthma medications [35,70]. Behbehani & Al-Yousifi (2003) calculated that the annual cost of a year’s supply of medications for a moderate asthma case was equivalent to US$ 562; cost of medications for a severe persistent case of asthma was found to be almost equivalent to the monthly salary of a nurse working in Kuwait [35]. Khadadah (2013), in a more recent study, estimated the annual cost of treating asthma cases among Kuwaiti nationals attending government health care facilities in Kuwait [70]. The estimated cost of treating asthma cases among Kuwaiti nationals was US$ 208 244 564, with the greatest cost drivers being inpatient hospital stays and emergency department visits, while medications constituted only 7% of total direct costs of treatment [70].

## DISCUSSION

As NCDs’ burden in the Arab region continues to grow, it becomes more necessary to assess the impact (financial and economic) of NCDs on patients and governments. In this review, studies providing quantification of costs associated with NCDs in 22 Arab countries, their treatment, management, or risk factors were included. The review identified and summarized only 55 studies covering the 16-year period (2000-2016). Costing studies were derived from LMICs like Sudan, Palestine, and Morocco, upper-middle-income countries and HICs, with four studies covering multiple countries in the Arab region [74-76,89]. All four classes of major NCDs [5], including diabetes, asthma, cancer and cardiovascular diseases were evaluated, and costs were determined for treatment or management of diseases, at the societal, governmental, provider, or patient level.

The studies were classified by costing variables such as costing approach, costing perspective, types of costs, and sources of information, although many of the studies did not indicate the method of costing used, nor specify the types of costs included. Furthermore, there was a large variation in the methods used to quantify NCDs’ costs in these countries. This lack of standardization made it difficult to conduct any type of cross-country, intra-country, or international comparisons. Any kind of cross-country comparison was further impeded by a focus, in the majority of identified studies, on treatment or management of only one class or type of NCD, with the exception of one study from Lebanon, which looked at costs of all smoking-related NCDs [89]. Also limiting cross-country and intra-country comparisons was inclusion of only one or a few variables of cost in calculations, with almost no calculations of the costs of NCDs covered in their totality. As such, it was not possible to identify trends in the costs of NCD management for Arab countries. Only three studies from Morocco used similar methodologies to quantify the costs of different classes of cancer to the Moroccan government [52,59,61]. These studies were also among the most comprehensive in their calculations, looking at different disease stages, and considering the incidence of the disease, and the different treatment modalities [52,59,61]. Even in the latter case, the heterogeneity in the cost calculation did not allow for trend identification. Nevertheless, the use of a semi-standardized method to quantify the direct costs of the different types of cancer in Morocco had its advantages. It allowed authors to make comparisons with international countries at an individual treatment level, allowed them to make comparisons to the Ministry of Health budgets, both at national and regional levels, and to make comparisons to national income levels [52,59,61]. In all cases, the direct cost of treatment was found to be higher than national budgets, higher than minimum income, but lower than the cost in countries used for comparison, pointing to the heavy burden that cancer treatment places on individuals and governments [52,59,61]. Such comprehensive results are useful for governments and decision-makers when allocating budgets and prioritizing funding to health facilities [52,59,61]. Yet studies from Morocco failed to look at cancer cost in its totality, and excluded crucial variables like indirect costs, productivity loss, and costs associated with outpatient treatment; therefore, costs obtained are likely an underestimation of the true cost of this NCD [52,59,61]. This was a common problem across most studies included in this review. Other methodological limitations identified from the studies included the use of different sampling frames and study designs, due to the epidemiological nature of the majority of the studies included. At the individual country level, instability, data scarcity, and struggling health care (information) systems could explain the variation in the data available to measure costs of NCDs, and thus the varying methodologies used [90,91].

The closest comparison to findings can be extracted from studies conducted in HICs, and from members of Organization for Economic Co-operation and Development (OECD). One such study looked at NCDs’ impact on national health expenditure [92]. Researchers found for the majority of included countries that NCDs accounted for at least one third of countries’ national health expenditure [92]. This analysis was possible because these countries, mostly OECD members, used a national health account framework for analysis [92]. The availability of standardized data on costs from these countries even made it possible to compare expenditure at two different time periods [92]. Among those studies identified in this review, few considered the time horizon when assessing the costs of NCDs, A systematic review that looked at NCDs’ global impact on health care spending and national income, mostly for countries in the American and European WHO regions, found that global health care expenditure on NCDs was increasing with time; furthermore, NCDs were resulting in national income losses [93]. However, this review only included one country from the Arab region [93]. For the most part, other reviews focusing on NCDs’ costs to individuals and households suffered from similar methodological limitations as those identified in this review [29,88].

### Limitations

Due to the fact that our study was part of a larger epidemiological approach scoping review, the included studies analyzed in this review are subject to several limitations including absence of a clear definition of costing method used, wide heterogeneity in methods followed to calculate same and different types of cost and variation in case definition. Other limitations are related to missing data on patient characteristics, which could have affected care or cost, sample representativeness like exclusion of individuals not seeking care for financial reasons and uneven geographical distribution. There are also differences between health systems in Arab countries, affecting the allocation of health funds for NCDs’ management. These factors did not allow us to pool reported cost estimates, to generalize results or to generate comparisons across studies. Another limitation is the search language used. This review only identified studies published in English, or containing an English abstract or keywords, potentially impacting number of studies identified and included in the review.

## CONCLUSIONS

The burden of NCDs in the Arab region is set to continue growing, conforming to local and global trends. This scoping review on the costs of NCDs in Arab region sheds light on an important issue: although NCDs-related morbidity and mortality continue to rise in all Arab countries across different income levels, data on costing remains limited by this type of evidence’s paucity and the generally low quality of studies published in this area. Internationally, NCDs resulted in high health care costs for governments and in great out-of-pocket and catastrophic health expenditures for households. Still, global findings and trends regarding NCDs raises questions of representativeness when inferring about applicability in the local and regional context. Moreover, even at international levels, questions persist concerning methodologies used for inferring costs at the national level.

Furthermore, although this review represents the most comprehensive to-date assessment of studies in the region directly quantifying the costs of NCDs, it remains restricted by the paucity of evidence and the generally low to very-low quality of included studies. Hence, if decisions are to be made based on available rough estimates, resources might be used inefficiently.

This research represents a foundational step for policymakers in need of evidence when managing the financial burden of NCDs in future reforms. There is also a need for future studies, of improved and harmonized methodology, from the Arab region on the cost management of NCDs and their growing financial impact at household and governmental levels.
