# Supplementary material for: Costs associated with management of non-communicable diseases in the Arab Region: a scoping review
Source: J Glob Health. 2018 Dec 10;8(2):020410. doi: 10.7189/jogh.08.020410 (PMC6287209; doi:10.7189/jogh.08.020410)
Supplement: Online Supplementary Document [file jogh-08-020410-s001.pdf]

Online Supplementary Document

Saleh et al. Costs associated with management of non-communicable diseases in the Arab Region: a scoping review

J Glob Health 2018;8:020410

Appendix S1:

Search Strategy

1 exp glucose metabolism disorders/ or exp diabetes mellitus/ or exp prediabetic state/ or exp glycosuria/ or exp lipid metabolism disorders/ or exp dyslipidemias/ or exp hyperlipidemias/ or exp hypercholesterolemia/ or exp hyperlipidemia, familial combined/ or exp hyperlipoproteinemias/ or exp hypertriglyceridemia/ or exp hypolipoproteinemias/ or exp lipidoses/ or exp metabolic syndrome x/ (503072)

2 (Diabet* or glucose* or ketone* or ketoacidosis or (Donohue adj syndrom*) or gylcosuria or hyperglyc?emi* or (glucose adj intoleran*)).tw. (761038)

3 ((insulin adj2 resistan*) or triglyceride or hyperlipidemi* or hdl or ldl or vldl or cholesterol or lipoprotein* or (lipid adj2 profile) or (lipid adj2 metabolism adj2 disorder*) or adiposit* or (abdominal adj fat) or hypercholesterolemi* or dyslipid?emi* or (blood adj lipid*) or hypertriglyceridemi* or hyperlipoproteinemi*).tw. (378968)

4 exp drinking behavior/ or exp alcohol abstinence/ or exp alcohol drinking/ or exp "tobacco use"/ or exp smoking/ or exp "tobacco use cessation"/ or exp smoking cessation/ or exp motor activity/ or exercise/ or exp physical fitness/ (416821)

5 diet/ or exp diet, diabetic/ or exp diet, carbohydrate-restricted/ or exp diet, cariogenic/ or exp diet, fat-restricted/ or exp diet, high-fat/ or exp diet, protein-restricted/ or exp diet, reducing/ or exp diet, sodium-restricted/ or exp diet, vegetarian/ or exp diet, western/ or exp energy intake/ (188024)

6 ((physical adj2 (activit* or inactivit*)) or exercise or exercising or (motor adj2 activit*) or bicycle or transport or walk or (intake adj2 (salt or sodium)) or (salt adj2 reduction) or tobacco or smoke* or smoking or argile or nargile or shisha or hookah or (second adj hand* adj2 smok*) or alcohol or beverage* or (drink* adj2 (binge or heavy)) or (drink* adj2 driv*)).tw. (1002273)

7 exp hypertension, pulmonary/ or exp asthma/ or exp bronchitis, chronic/ or exp pulmonary disease, chronic obstructive/ or exp pulmonary emphysema/ (178424)

8 ((chronic adj respiratory) or CRD or COPD or (chronic adj obstructive adj pulmonary) or emphysema or (occupational adj lung) or (pulmonary adj hypertension) or asthma* or (respiratory adj2 allerg*) or (respiratory adj hypersensitivit*)).tw. (216572)

9 exp cardiovascular diseases/ or exp cardiovascular abnormalities/ or exp cardiovascular infections/ or exp heart diseases/ or exp pregnancy complications, cardiovascular/ or exp vascular diseases/ (2036533)

10 (Cardiomegal* or cardiomyopath* or ((heart adj disease*) or disorder* or failure*) or arrhythmia* or tachycardia* or endocard* or myocard* or arrest* or diastolic or systolic or (myocardial adj2 infarction) or isch?emi* or (angina adj2 pectoris) or shock or aneurysm or angiopath* or embolism or thrombosis or hypertension or (blood adj pressure) or phlebitis or atherosclerosis or arteritis or (vascular adj malformation) or (arteriovenous adj malformation) or ((coronary adj disease*) or disorder*)).tw. (2822581)

11 exp Neoplasms/ (2813163)

12 (Neoplasm* or cyst* or tumo?r* or cancer* or carcinoma or carcinoid or malignan* or metastat* or melanoma or sarcoma or leuk?emia or leuc?emia or lymphoma or angiosarcoma or astrocytoma or glioma or Cholangiocarcinoma or Chondrosarcoma or Craniopharyngioma or Ependymoma or Fibrosarcoma or hemangioendothelioma or Leiomyosarcoma or Liposarcoma or Medulloblastoma or Meningioma or Mesothelioma or Neuroblastoma or Oligodendroglioma or Osteosarcoma or Pheochromocytoma or Pineoblastoma or Rhabdomyosarcoma or Thymoma or adenocarcinoma).tw. (3065498)

13 or/1-12 (8724680)

14 cost-benefit analysis/ or "cost of illness"/ or health care costs/ or health expenditures/ (116890)

15 Feasibility Studies/ (49650)

16 Insurance, Health, Reimbursement/ec, sn [Economics, Statistics & Numerical Data] (2459)

17 Health Resources/ec, sn [Economics, Statistics & Numerical Data] (2271)

18 Insurance Coverage/ec, sn [Economics, Statistics & Numerical Data] (3522)

19 "Fees and Charges"/ (8617)

20 (cost* or feasibl* or (health adj resourc*) or financ* or payment or reimburs* or coverage or charge or expense or (health adj expenditur*) or (resource adj investment)).tw. (794640)

21 or/14-20 (878363)

22 (Egypt or Algeria or Sudan or Iraq or Morocco or Saudi Arabia or Yemen or Syria or Tunisia or Somalia or United Arab Emirates or Libya or Jordan or Palestine or Lebanon or Mauritania or Kuwait or Oman or Qatar or Bahrain or Djibouti or Comoros).tw. (57328)

23 13 and 21 and 22 (846)

24 limit 23 to yr="2000 - 2016" (725)
